# Comparative analyses of the IV group oxides additives influence on the sintering kinetics of zirconia nanopowders

**DOI:** 10.1371/journal.pone.0200869

**Published:** 2018-07-27

**Authors:** Marharyta Lakusta, Igor Danilenko, Galina Volkova, Larisa Loladze, Valeriy Burchovetskiy, Tetyana Konstantinova

**Affiliations:** Material Science Department, Donetsk Institute for Physics and Engineering (DIPE) named after O.O. Galkin of the NAS of Ukraine, Kiev, Ukraine; University of Vigo, SPAIN

## Abstract

Ceramics materials based on yttria stabilised tetragonal zirconia nanopowders (3Y-TZP) are widely used in the production fuel cells, oxygen sensors, refractories, etc. and intensively studied due to their outstanding mechanical and electrical properties. To obtain ceramics with specific properties different additives can be used. This allow for control over the features of a nanopowder’ structure and its consolidation during the sintering process. It is important examine in detail the initial sintering stage of tetragonal zirconia based nanopowders modified by third element additives. The present paper shows the impact of SiO_2_ and SnO_2_ additives and the influence of different methods of dopant addition (co-precipitation and mechanical mixing) on the kinetics of the initial sintering stage of tetragonal zirconia nanopowders. It demonstrates that sintering mechanism is changed by the addition of a small amount SiO_2_ and SnO_2_ by volume to the grain boundary diffusion in 3Y-TZP nanopowders obtained by the co-precipitation method. This change led to increased activation energy. This paper explores the reasons for the acceleration in the sintering process of nanopowders 3Y-TZP with SiO_2_ and SnO_2_ additives obtained by the mixing method. It shows that the sintering mechanism is the same as that of the initial 3Y-TZP powder obtained by the co-precipitation technique. The volume diffusion mechanism was the predominant mechanism at the initial sintering stage and was accompanied by a decrease in the activation energy of the sintering process.

## Introduction

Nanomaterials based on yttria stabilised zirconia with the addition of third oxide components and oxides of rare earth elements in tetragonal and cubic crystal modifications have a number of unique physical and mechanical properties and have already found significant applications as solid electrolytes of high-temperature electrochemical devices [1–5], various structural ceramics, cutting tools and abrasive materials. It is well known that zirconia based materials are an excellent for use in medicine and dentistry [5–7].

Many different methods of obtaining nanopowders have been developed such as co-precipitation from salt solutions, hydrolysis, spray pyrolysis, the sol-gel process, the aerosol method, laser synthesis, the mechanochemical method, flame treatment, chemical gas-phase condensation, etc. [7–9]. A comparison of these methods for obtaining zirconia nanopowders has identified the simplest and cheapest methods—the co-precipitation technique and the mechanical mixing method, respectively, which were used for the production of nanopowders in the present study [9–10].

The main advantage of the co-precipitation method is its ability to dope zirconia with different elements (Ni, Si, Ge, Cr, Al, etc.) during the main processing stage without additional costs and procedures [10–17]. The possibility of obtaining composite materials during a single technological stage opens great prospects for this method. Earlier it was shown that composite materials of 3Y-TZP-2 wt % Al_2_O_3_, sintered from nanopowders obtained by the co-precipitation method, led to a significant increase in the material’s crack and wear resistance [17–18].

A small number of papers have been devoted to the study of the sintering kinetics of zirconia based nanopowders with different additives. The initial stage of zirconia powders densification during the sintering processes was studied by several researchers: Matsui [12–18] investigated Tosoh produced powders with SiO_2_, GeO_2_, Al_2_O_3_ addition, Sakka [17] examined Tosoh powders with Al_2_O_3_ additive, processed with ultrasound and Maca [18] studied powders 3Y-TZP. All this authors have found that the mechanism of grain-boundary diffusion is the dominant sintering mechanism in 3Y-YTZP nanopowders without any dopants. In papers [12–18] the predominant sintering mechanisms at the initial sintering stage of 3Y-TZP have been studied. It was shown that a small amount of various oxides, for example Matsui reported that addition of 0.25 wt% SiO_2_ or GeO_2_, led to a change in the sintering mechanism from grain boundary diffusion to the volume diffusion mechanism with the shrinkage beginning at temperatures 50–80 °C lower, than in the starting powders without additives. At the same time, the activation energy of the sintering process decreased. Matsui explained the decrease in the activation energy and the acceleration of the densification rate by the partial solubility of alumina, silica and germanium oxide in zirconia, although Matsui indicates that their solubility is low. In their papers [14–17] the comparative analyses of the influence of different additives type on the sintering mechanisms of zirconia nanopowders did not carry out. In addition to this fact neither the methods of the nanopowders’ synthesis nor the additive types have been taken into account. Therefore, we can assume that the diffusion mechanism of 3Y-ZP at the initial sintering stages could be determined by them incorrect and this take additional research.

In our previous studies [19–21] we have studied the sintering kinetics of tetragonal zirconia nanopowders. It has been established that the initial 3Y-TZP nanopowder was sintered due to the predominant of volume diffusion mechanism that completely contradicts the results obtained by other researchers which have reported that the grain boundary diffusion mechanism was the dominant sintering mechanism [20–21]. It became interesting to investigate the reason of such inconsistent results in the sintering mechanisms in powders with equal characteristics. We supposed that it caused by the difference in powders preparation methods which effected on the diffusion mechanism that was not taken into account by the authors of the papers [12–18].

Studies on nanopowders, due to their nature usually focus on the initial sintering stage because it enables researchers to examine the mechanisms of nanoparticles’ interactions upon heating and to determine the influence of particle size, methods of preparation and other characteristics of the starting materials on these mechanisms. As noted earlier, other additives such as SiO_2_, GeO_2_, Al_2_O_3_ have a similar effect on the sintering mechanisms in 3Y-TZP, although the physical characteristics of these additives are different. For example, their melting points are 1600°, 1115° and 1326 °C respectively and differ significantly from the melting point of Al_2_O_3_ (2072 °C) and their ionic radiuses are different (Si^4+^—0.039 nm, Sn^4+^—0.069 nm, Al^3+^—0.062 nm). As stated above, the change in sintering mechanisms was explained by Matsui as the dissolution of all segregated additives on the surface of zirconia without relation to dopant types and we supposed that this explanation is partially incorrect and require more thorough study.

Based on the above, we consider that it is important to research the influence of different synthesis methods and different additives types on the kinetics of the initial sintering stage of tetragonal zirconia and to reveal their effect on the mass transfer mechanisms during sintering.

The aim of the present paper is to carry out the comparative investigation the influence of the small amounts of SiO_2_ and SnO_2_ additives and to study the influence of different synthesis methods on the diffusion mechanism at the initial sintering stage of tetragonal zirconia nanopowders (3Y-TZP).

## Methods

The investigated nanopowders: ZrO_2_+3 mol% Y_2_O_3_ (3Y-TZP), ZrO2+3 mol% Y_2_O_3_+n∙SiO_2_ (3Y-TZP+ n∙SiO_2_), ZrO2+3 mol% Y_2_O_3_+n∙SnO_2_ (3Y-TZP+n∙SiO_2_) were obtained by co-precipitation method. Dopants concentration is n = 0.2 and 2 wt%. 3Y-TZP was synthesized using ZrOCl_2_⋅nH_2_O, Y(NO_3_)_3_⋅nH_2_O salts. The preparation technique was described in detail in papers [12, 20–22]. 3Y-TZP with similar concentration of SiO_2_ and SnO_2_ additives were also obtained by mixing method. Abbreviation PMM8 was marked for powders obtained by mixing with milling for 8 hours PMM8-ZrO2+3 mol% Y_2_O_3_+n∙SiO_2_ (PMM8-3Y-TZP+ n∙SiO_2_), PMM8-ZrO_2_+3 mol% Y_2_O_3_+n∙SnO_2_ (PMM8-3Y-TZP+n∙SiO_2_).

In the present paper the nanopowder’ compositions 3Y-TZP- 0,2 wt % SiO_2_ and PMM8-3Y-TZP- 0,2 wt % SiO_2_ were not considered in detail because this powders were investigated in our previous study [22]. In the present paper we compare the results obtained in paper [22] with results obtained on 3Y-TZP based powders’ described above with different additive—SnO_2_ and with different concentration of SiO_2_ -2wt%. After precipitation finalization the synthesized hydrogels were dried in a microwave furnace with an output power of 700 W and at a frequency of 2.45 GHz. The dried zirconium composites hydroxides were calcined in a resistive furnace at 1000 °C with a dwelling time of 2 h to compare our results with data obtained by other researchers who used the Tosoh powders with similar initial zirconia particle sizes. Then calcined nanopowders 3Y-TZP, 3Y-TZP-SiO_2_, 3Y-TZP-SnO_2_ were mechanically milled in a planetary mill MSK-SFM-1 (MTI Corp., USA) at 400 rpm for 8 h using YSZ milling balls.

Thereafter, all nanopowders were pressed at 300 MPa and sintered up to the temperature of 1500 °C with different heating rates of 2.5°, 5°, 10°, 20 °C/min in the dilatometer (NETZSCH DIL 402 PC). The shrinkages data of all samples during sintering were obtained by a dilatometry method. Thermal expansion of each sample was corrected with the cooling curve by the method described in [12, 15–16]. The final density of sintered samples was measured using the Archimedes method. The parameters of all nanopowders were investigated by X-ray diffraction (XRD) employing the Dron-3 diffractometer with Cu-K α radiation. Fitting and analysis of the XRD curves were made by Powder Cell software for Windows version 2.4. Particle sizes (d_XRD_) were calculated using the Debay–Scherrer Equation. The specific surface areas were measured by the Brunauer-Emmett-Teller (BET) method using “SORBI-4” device. The particle sizes and powders structures were studied by the transmission electron microscope TEM (Jem 200A, JEOL, Japan). The microstructures of the ceramics were studied by scanning electron microscopy (JSM 6490LV JEOL) after polishing and thermally etching (at 1450 °C during 0.5 h) of the surfaces. The chemical composition and elementary mappings of sintered materials were checked by the energy dispersive spectroscopy (EDS) analysis (Inca Oxford, England). The particle’ sizes were determined from the measurements of 200–250 particles in TEM images using the standard secant method.

To analyze the obtained dilatometric data was used the standard constant rate of heating (CRH) technique [22–24]. This analytical method is applicable only for analyzing the initial sintering stage (when the relative shrinkage is not more than 5%). The sintering-rate equation at the initial sintering stage is given by the following equation which was derived by Wang and Raj equations [23, 24]:

$$ln[T(\frac{dT}{dt})(\frac{d\rho }{dT})]=-\frac{Q}{RT}+ln[f(\rho )]+lnA-Nlnd$$ (1)

here, T—the temperature; dT/dt—the heating rate; ρ—the density; Q—the activation energy; R—the gas constant; F(ρ)—the density function that depends on n.

Using the slope S_1_ of the Arrhenius-type plot of ln[T(dT/dt)(dρ/dT)] against 1/T, the Q is expressed as

$$Q=-RS_{1}$$ (2)

The sintering parameter n was determined using the Matsui’s equations [14] which were derived from the Yang and Cutler’s equations [24]:

$$\frac{d(\Delta L/L_{0})}{dT}={\left(\frac{2.14\gamma \Omega bD_{0B}RT}{ka^{3\;}cQ}\right)}^{\frac{1}{3}}(\frac{Q}{3RT^{2}})exp(-\frac{Q}{3RT})$$ (3)

$$\frac{d(\Delta L/L_{0})}{dT}={\left(\frac{5.53\gamma \Omega bD_{0v}RT}{ka^{3\;}cQ}\right)}^{\frac{1}{2}}(\frac{Q}{2RT^{2}})exp(-\frac{Q}{2RT})$$ (4)

here ΔL = (L_0_-L) is the change in length of the specimen; c = dT/dt is the heating rate and D_0_ is the pre-exponential term defined as D = D_0_exp(-Q/RT); K-the numerical constant; g -the surface energy; V- the atomic volume; D -the diffusion coefficient, t—the time, T -absolute temperature; k -the Boltzmann’s constant; a -the particle radius; and the parameters n and p are the order depending on the diffusion mechanism.

Using the slope S_2_ of Arrhenius-type plot of ln[T^2-n^ d(ΔL/L_0_)/dT] against 1/T the apparent activation energy (nQ) is expressed as:

$$nQ=-RS_{2}$$ (5)

Considering that if n = 1, this means that the viscous flow mechanism dominates. If n = 0.4–0.5, the volume diffusion mechanism dominates and if n = 0.3–04, the grain boundary diffusion mechanism dominates [12–18].

## Results and discussion

Table 1 shows all characteristics of the investigated 3Y-TZP nanopowders taking into account of obtaining methods, additive types and concentrations.

**Table 1:** The X-ray and BET analysis results.

Nanopowders composition	Coherent scattering area, nm	The phase composition, % M-phase	S_BET_ g/m^2^	Lattice parameters, a and c, Å **
**3Y-TZP**	31.5	5%M + T	14	a = 5,0980, c = 5,17396
**3Y-TZP+2 wt % SiO**_**2**_	26	100% T	20	a = 5,09126, c = 5,17582
**3Y-TZP+0,2 wt % SnO**_**2**_	31.5	8%M+T	13	a = 5,09629, c = 5,16165
**3Y-TZP+2 wt % SnO**_**2**_	29.2	4% M%+T	17,3	a = 5,09321, c = 5,164681
**PMM8-3Y-TZP**	29	15,5%M+T	19.9	a = 5,095163, c = 5,16670
**PMM8-3Y-TZP+ 2 wt % SiO**_**2**_	26.5	12%M+T (+SiO_2_)	20.4	a = 5,0946, c = 5,16561
**PMM8-3Y-TZP+0,2 wt % SnO**_**2**_	28	7%M+T	17.6	a = 5,0950, c = 5,16659
**PMM8-3Y-TZP+2 wt % SnO**_**2**_	27.5	9%M+T (+SnO_2_)	17.9	a = 5,0941, c = 5,16657

M-the monoclinic phase amount,

T- the tetragonal phase amount.

**Standard deviation of the lattice parameters determination is Δa = ±0,0006, Δc = ±0,0005.

**Fig 1:**
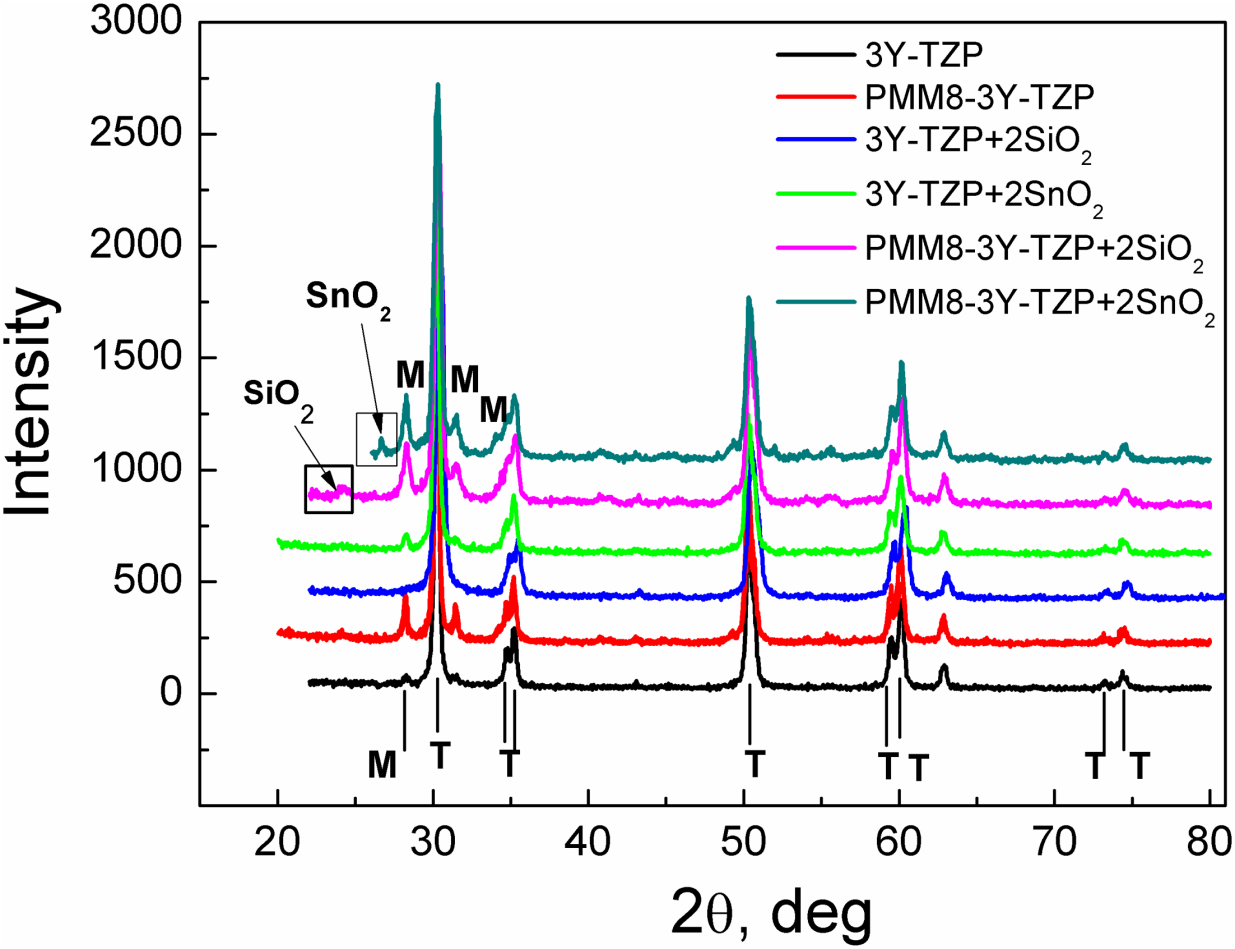
The XRD patterns of nanopowders with and without additives: 3Y-TZP; PMM8-3Y-TZP; 3Y-TZP+2 wt% SiO_2_; 3Y-TZP+2 wt% SnO_2_; PMM8-3Y-TZP+2 wt% SiO_2_; PMM8-3Y-TZP+2 wt% SnO_2_.

The XRD spectra of all investigated nanopowders synthesized by the mixing and co-precipitation techniques are presented in Fig 1. The additives and 8 hours of milling hardly affected the crystallites’ size and the phase composition of the nanopowders. Fig 1 confirms the fact that SiO_2_ and SnO_2_ additives were not found as a separate phase (the additives’ peaks should be situated within 25–27°of the XRD pattern).

**Fig 2:**
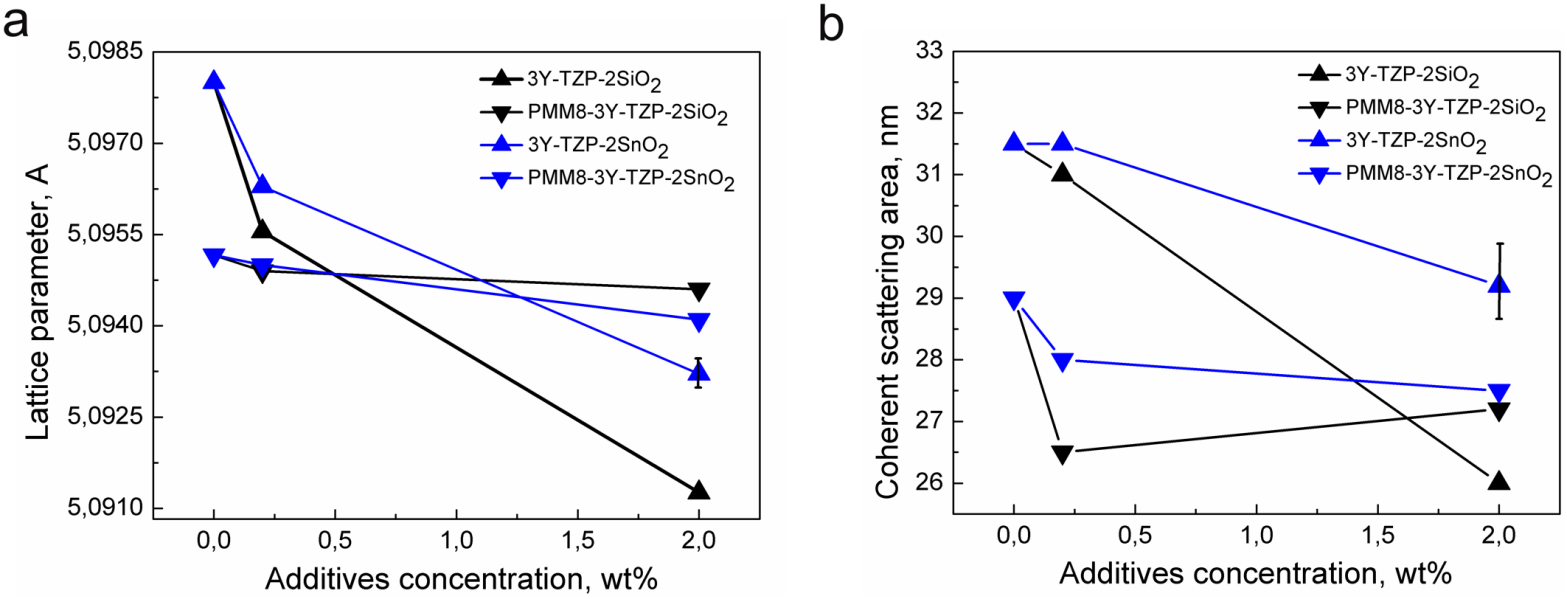
The lattice parameters (a) and the particle sizes (b) changing of the nanopowders obtained by co-precipitation (3Y-TZP, 3Y-TZP-SiO_2_, 3Y-TZP-SnO_2_) and by mechanical mixing (PMM8-3Y-TZP, PMM8-3Y-TZP-SiO_2_, PMM8-3Y-TZP- SnO_2_).

Fig 2 shows the changes in 3Y-TZP lattice parameters and particle sizes with the addition of dopants. It can be seen that SiO_2_ and SnO_2_ added by co-precipitation had a greater effect on the change in particle size and the lattice parameters of zirconia than the same dopants added by mechanical mixing. On the basis of these results (Fig 1, Table 1) it can be concluded that 3Y-TZP powders with additives of SiO_2_ and SnO_2_ obtained by the co-precipitation method created the supersaturated solid solution with the zirconia matrix.

The phase composition of 3Y-TZP only changed in the case of nanopowders that were milled mechanically during mixing. PMM8-3Y-TZP nanopowder without additives had the maximal monoclinic phase amount, reaching 15.5% after mechanical activation during 8 hours (Fig 1). The 8 hours of milling led to decrease in the lattice parameters of 3Y-TZP (Fig 2a, Table 1). It well known that mechanical activation leads to an increase in the monoclinic phase amount [1–2, 25].

**Fig 3:**
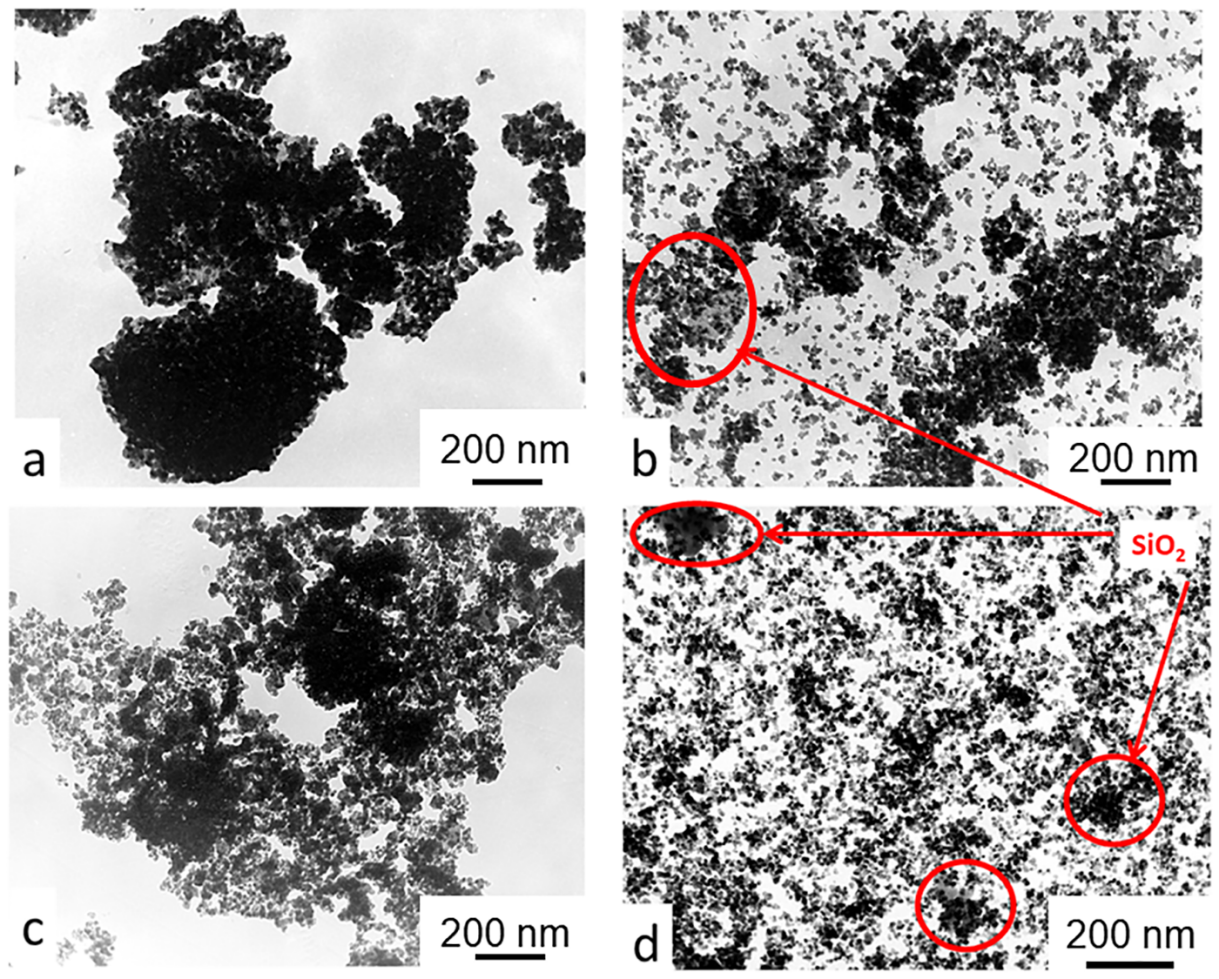
TEM images of (a) 3Y-TZP, (b) PMM8-3Y-TZP, (c) 3Y-TZP+2 wt%SiO_2_ and (d) PMM8-3Y-TZP+2 wt%SiO_2_ nanopowders.

With mechanical addition of the SiO_2_ and SnO_2_, the monoclinic phase amount decreased (Table 1) in comparison with milled 3Y-TZP powder. The peaks of the SiO_2_ and SnO_2_ can be seen on the XRD patterns of PMM8-3Y-TZP-SiO_2_/SnO_2_ (Fig 1), which confirms the fact that in cases of mechanical mixing, additives are detected as separate phases. This finding was confirmed by the TEM images (Fig 3d).

In the case of mechanically milled 3Y-TZP nanopowders with small amounts of additives, the decrease in the lattice parameters was only caused by milling (Fig 2a) because no solid solution was created in these composite nanopowders. The particle sizes decrease (Table 1, Fig 2b), the specific surface area increases. The additive with the most significant influence on specific surface area was observed to be a silica dopant added using the co-precipitation technique. With the addition of 0.2 wt% SiO_2_ it was 14.1 g/m^2^ [21] and with 2 wt% SiO_2_ it was 20 g/m^2^.

**Fig 4:**
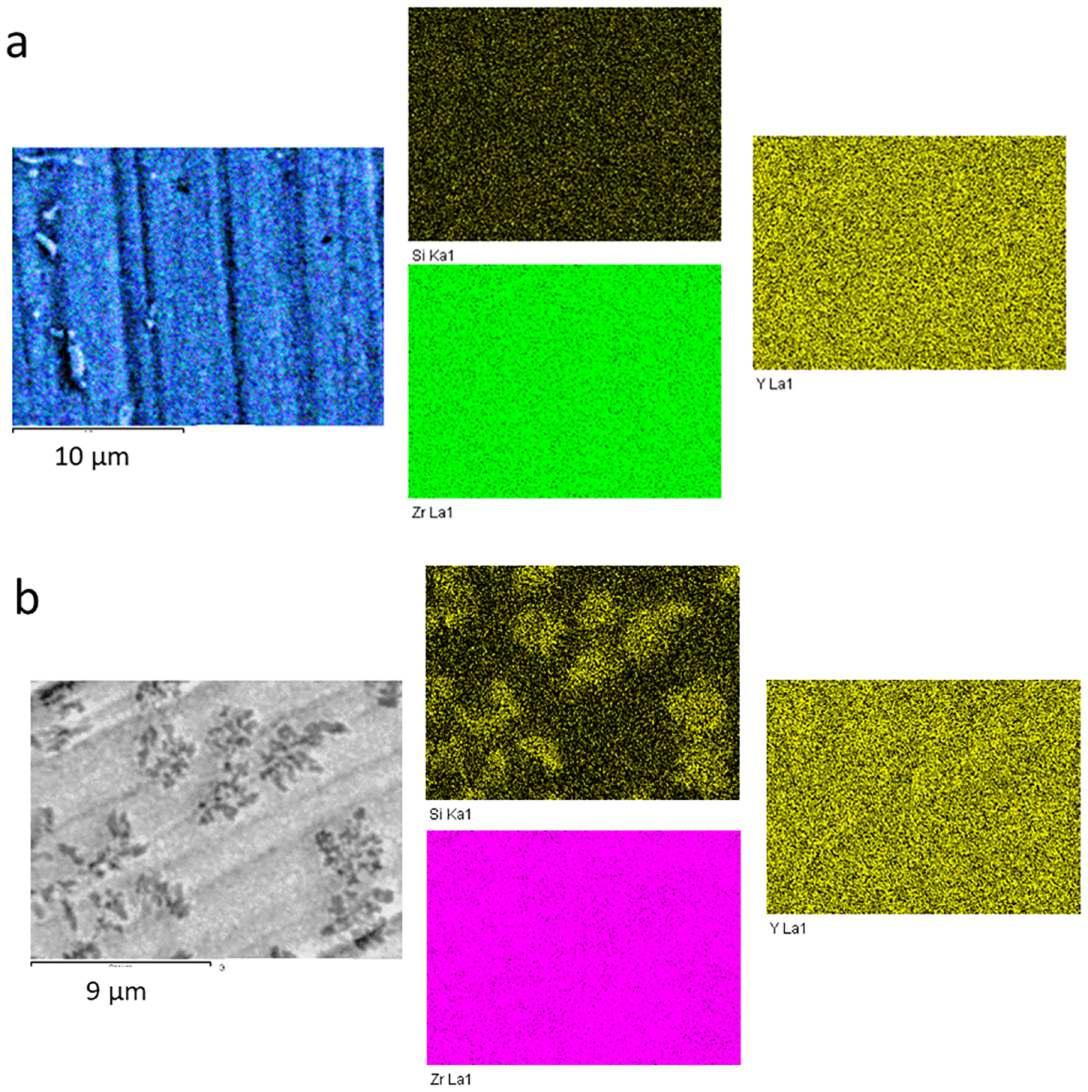
The elementary mapping of nanopowders 3Y-TZP-2 wt% SiO_2_, PMM8-3Y-TZP-2 wt% SiO_2_.

Fig 3 shows TEM images of 3Y -TZP nanopowders with and without a silica additive. As can be seen, the nanopowder 3Y-TZP (Fig 3a) has a sufficiently high degree of aggregation. However, it should be noted that aggregates are "soft" and can be easily destroyed by a mechanical action, observed after 4 hours [21] and after 8 hours of milling (Fig 3b). An important result obtained in our previous paper [19–21] showed that 8 hours of milling were enough to create a uniform additive distribution between the particles of the 3Y-TZP although it was noted that the addition of silica by mechanical mixing, was considered by XRD and TEM analysis to be a separate phase (Figs 1 and 3). Fig 3 illustrated the uniform distribution of the 2 wt% SiO_2_ additive in the 3Y-TZP powder’s structure, dependent on the dopant addition method. In Fig 4 are shown the elementary mappings of the nanopowders 3Y-TZP-2 wt% SiO_2_, PMM8-3Y-TZP-2 wt% SiO_2_ that confirm the different additives distribution in 3Y-TZP nanopowders structure.

The silica additive led to an increase in particle size, and this is confirmed by the XRD and BET analyses presented in Table 1 (Fig 2). The tin oxide additive behaves in a similar way when added to the 3Y-TZP. However, the effect of the SnO_2_ was not as significant as the influence of the silica. This is probably due to both its larger of ionic radius value (r (Sn^4+^) = 0.67 nm) and the absence of phase transformations during heating, in contrast to silica in which the phase transformation occurs during calcination at temperatures up to 1000 °C the phase transformation occurs during calcination at temperatures up to 1000°C and the ionic radius is much smaller than the radius of zirconium (r (Si^4+^) = 0.39 nm, r (Zr^4+^) = 0.0720 nm). The SiO_2_ additive affects the structure of the lattice as follows. At 1000°C—α-quartz has a hexagonal lattice and a very small ion radius. Silicon has a high chemical affinity for oxygen, and silicon oxides are stable. Probably due to the unfavourable ratio of the ion radius of Zr and Si, the silicon ions that have replaced zirconium ions at some nodes cause a nonisotropic interaction on the coupling forces in the three-component system, accompanied by a decrease in the lattice parameter *a* and an increase in parameter *c*. Parameter *c* increases faster than *a*, which leads to an increase in the tetragonality degree without changing the cell volume.

The complex of XRD, TEM and BET analyses of the obtained data prove that additives led to a modification of the 3Y-TZP nanopowders’ structure depending on the type and concentration of the additives and the method by which they were added. It is known that if the structure of the initial powder changes, the sintering parameters also change. Therefore, knowing the structure-property relationship, it becomes possible to obtain materials based on zirconia with desired properties.

**Fig 5:**
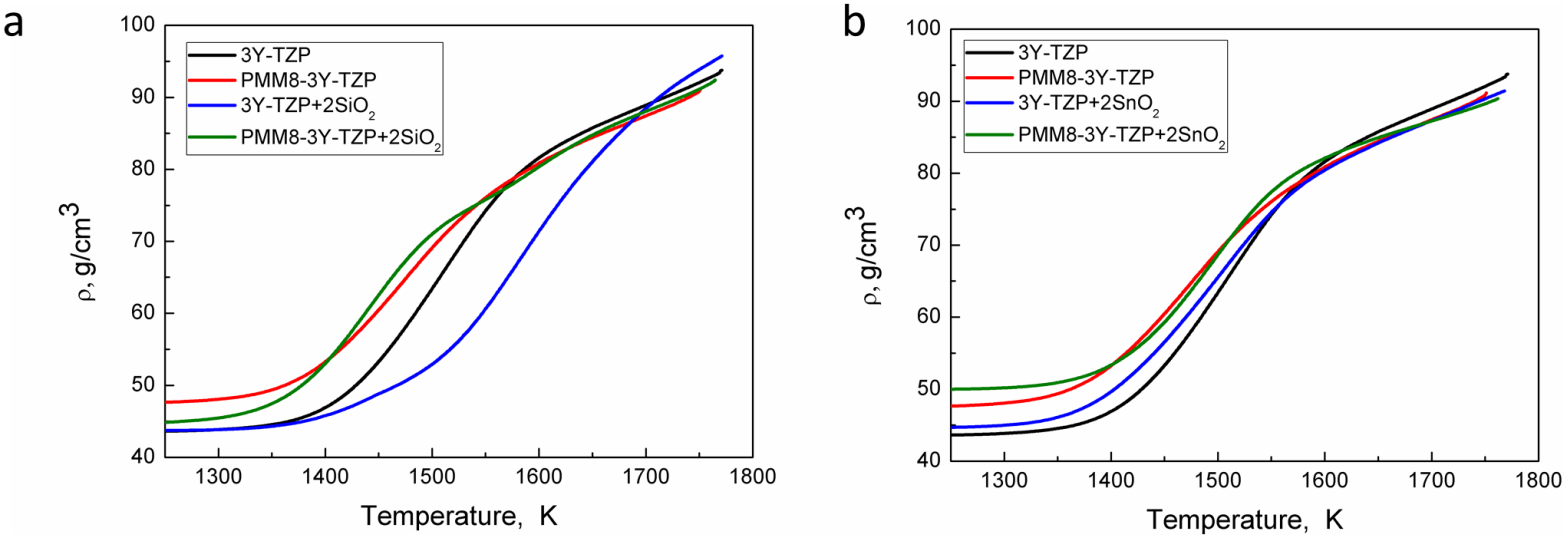
Relative density vs temperature for nanopowders system of (a) 3Y-TZP- SiO_2_ and (b) 3Y-TZP- SiO_2_/SnO_2_ (heating rate is 5 °C min^-1^).

**Fig 6:**
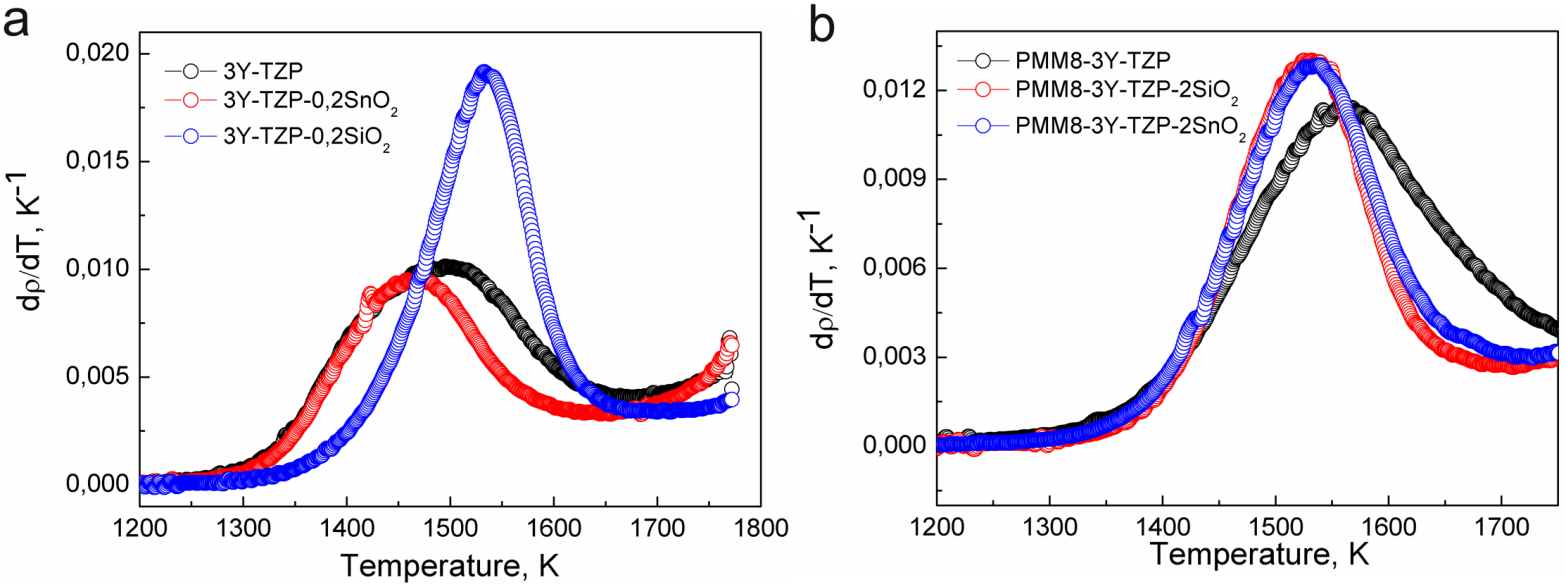
Temperature dependence of densification rates of the nanopowders obtained by co-precipitation (a) 3Y-TZP, 3Y-TZP+0.2wt% SiO_2_/SnO_2_ and by mixing (b) PMM8-3Y-TZP, PMM8-3Y-TZP+2 wt% SiO_2_/SnO_2_ at sintering rate 10 °C/min.

The relative density vs temperature is shown in the Fig 5 and the sintering kinetics behavior is shown in the Figs 5 and 6 (for nanopowders systems 3Y-TZP- SiO_2_ and 3Y-TZP- SiO_2_/SnO_2_ and densification rate). The temperature dependence of densification rates (dρ/dT) of the 3Y-TZP nanopowders with and without milling is shown in Fig 6. The nanopowder 3Y-TZP without milling achieved the maximum densification rate at a lower temperature (1500 K) than the milled nanopowders (1570 K). The densification curves for these powders shifted to the higher temperature.

The densification kinetics of the investigated nanopowders dependent on the synthesis method is shown in the Fig 6. The figure shows that a small amount (0.2 wt%) of SiO_2_ and SnO_2_ additives can have a significant influence on the dandification rate of 3Y-TZP nanopowders.

The temperature value of maximum densification rate (dρ/dT) in the co-precipitated powders (Fig 6a) shifted upwards the addition of the SiO_2_ and did not change when SnO_2_ was added. The opposite was observed for the samples obtained by mechanical mixing. The SiO_2_ addition led to a decrease in the temperature value of the maximum densification rate in contrast to the effect of the SnO_2_ additive.

**Fig 7:**
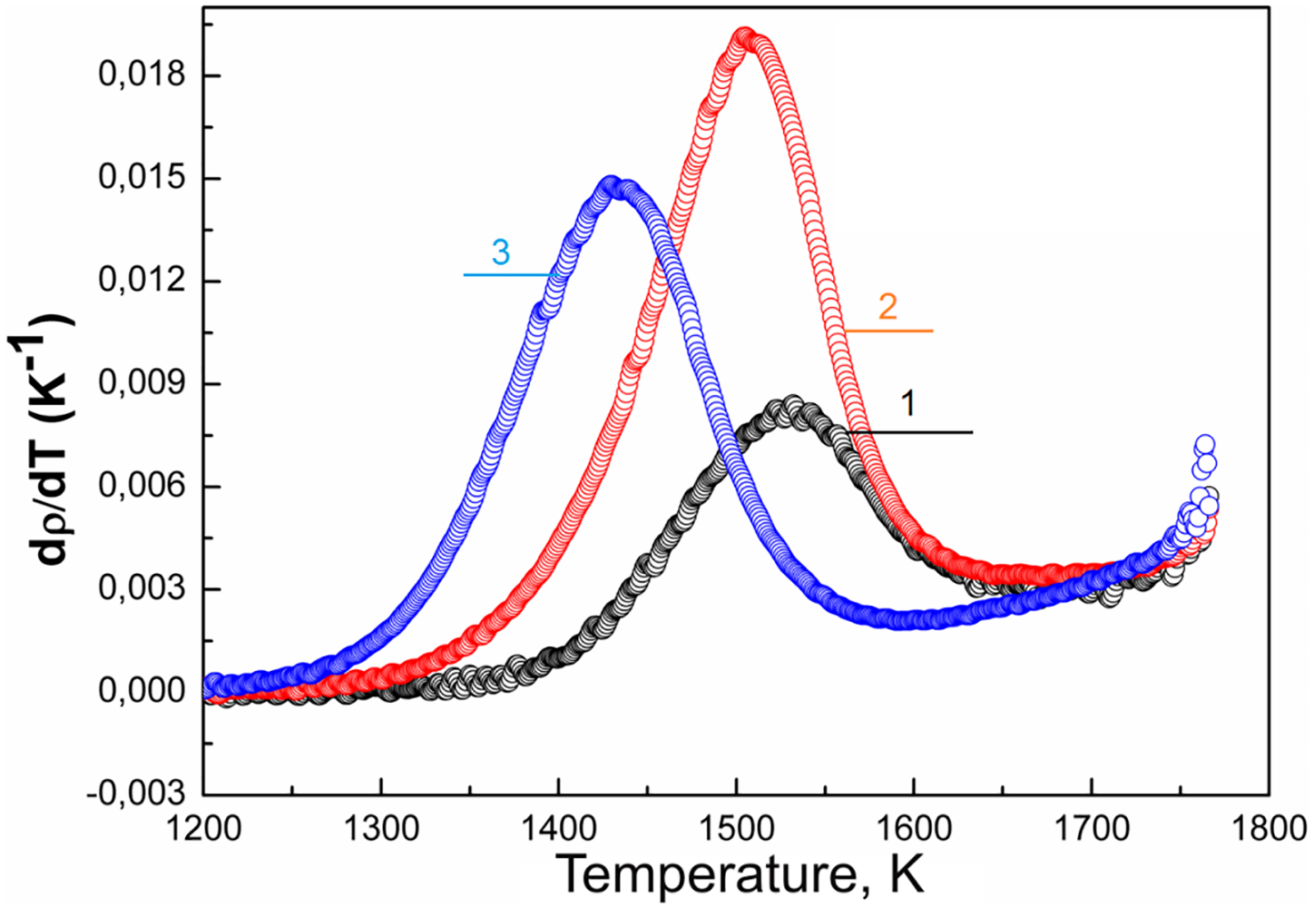
Temperature dependence of densification rates of the nanopowders with silica additive obtained by mixing (1) PMM8-3Y-TZP, (2) PMM8-3Y-TZP+0.2wt%SiO_2_, (3) PMM8-3Y-TZP+2wt%SiO_2_ at sintering rate 2,5 °C/min.

The influence of the additives’ concentration on the densification of 3Y-TZP is shown in Fig 7 with example of the silica additive. As the amount of the additive increased, the temperature value of the densification rate maximum shifted to the lower range. This may indicate that a change in the sintering mechanism has occurred. The opposite behaviour of shrinkage rate curves was investigated in an earlier study and described in our previous article [21].

**Fig 8:**
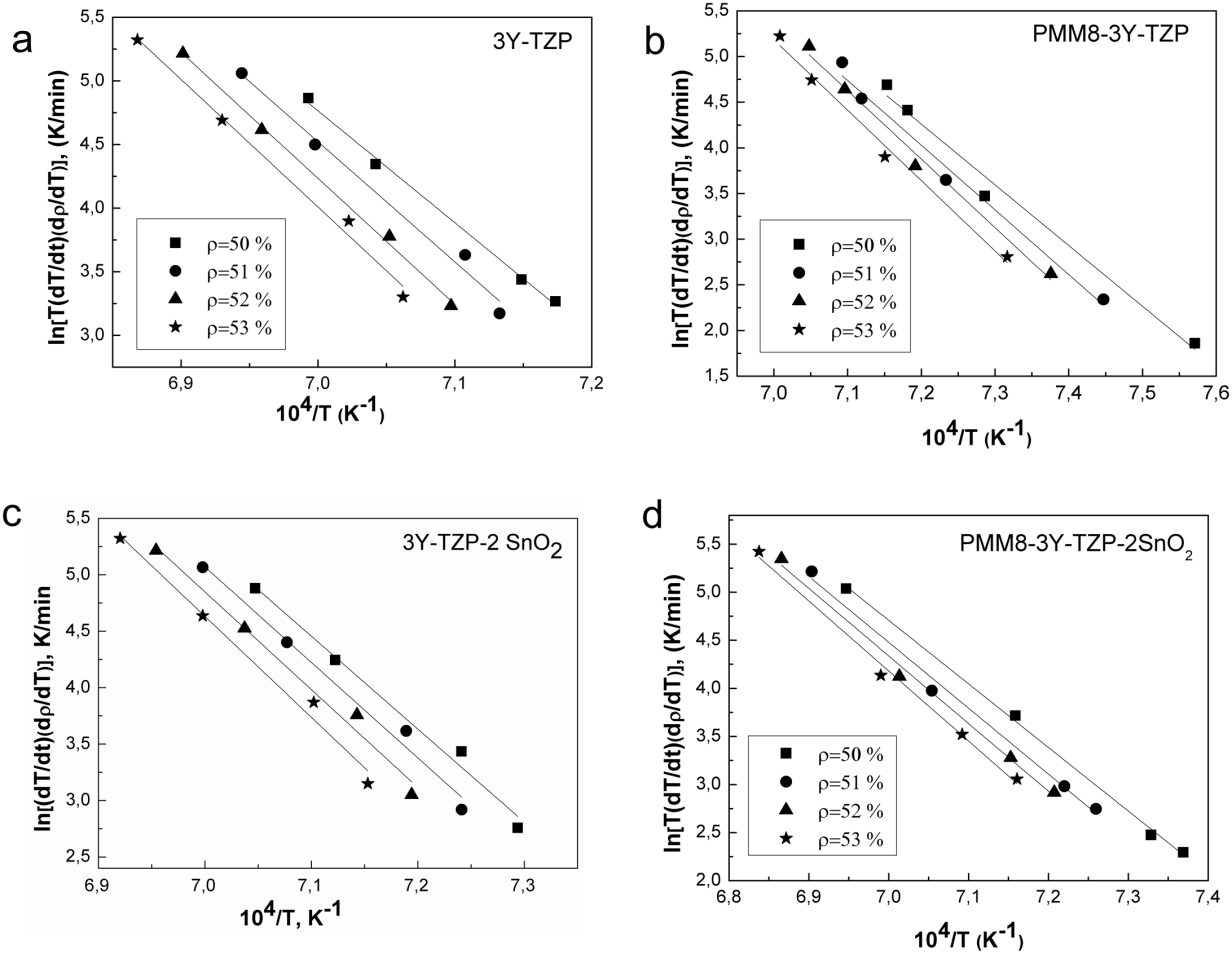
Arrhenius-type plots of 3Y-TZP nanopowders with and without additives.

**Fig 9:**
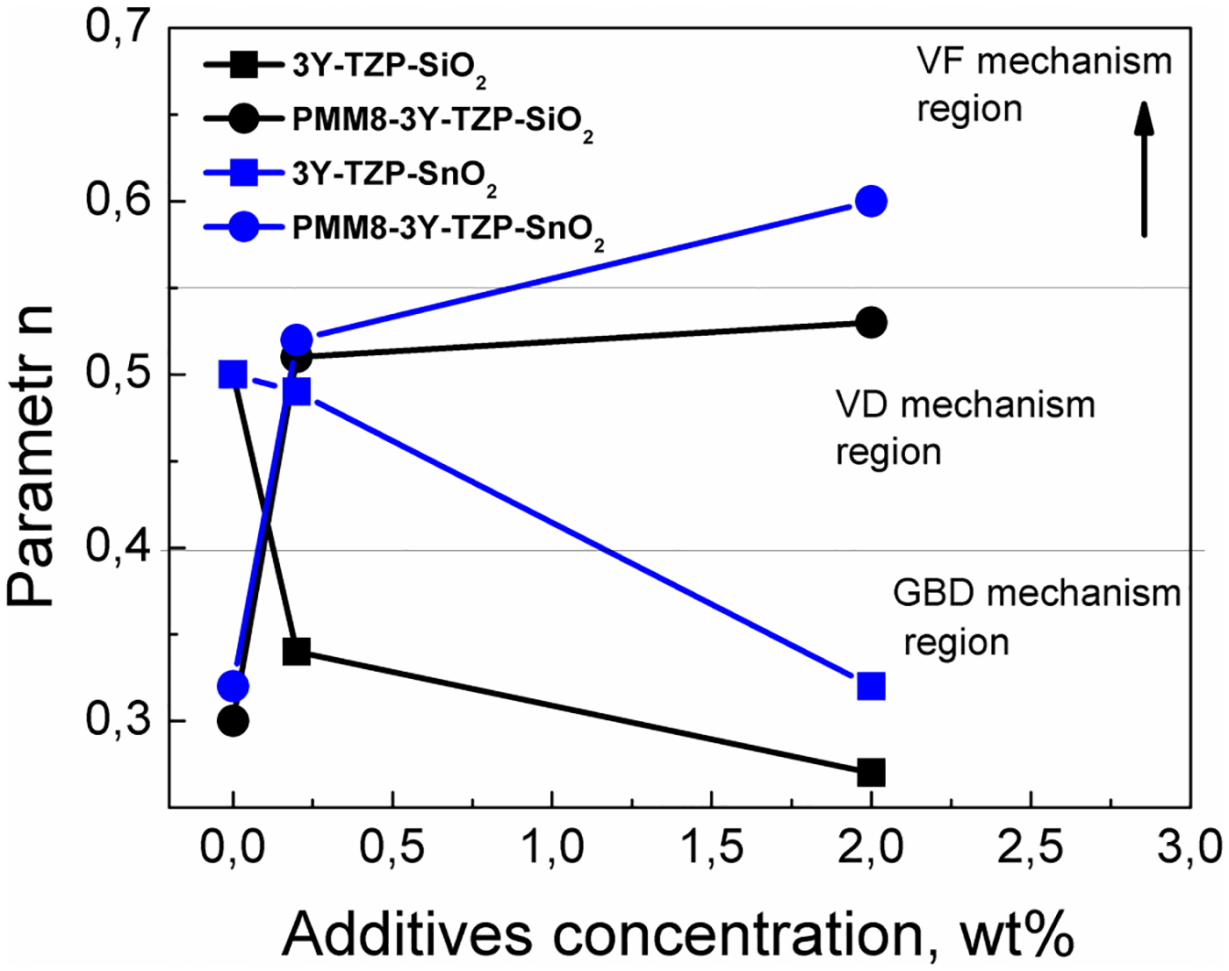
The sintering mechanism changing in depends on additive type and obtaining method of 3Y-TZP with SiO_2_ and SnO_2_ additives.

Using the analytical constant rate of heating method and Arrhenius-type plots (Fig 8), the sintering mechanisms that dominate at the initial sintering stage in all investigated nanopowders were determined as shown in papers [19–21]. In the Table 2 the results of the calculated dilatometry date (parameter n, activation energy Q and sintering mechanism that dominates at the initial sintering stage) for all investigated nanopowders are presented. The Fig 9 illustrates the changes in the sintering mechanism according to additive type and obtaining method of 3Y-TZP with SiO_2_ and SnO_2_ additives.

**Table 2:** The sintering parameter n, the activation energy of sintering Q and the sintering mechanism of all investigated samples.

Nanopowders	n	Q (kJ/mol)	Sintering mechanism
**3Y-TZP**	0,5	665±40	VD
**3Y-TZP-2 wt% SiO**_**2**_	0,28	720±40	GBD
**3Y-TZP-0,2 wt% SnO**_**2**_	0,49	741±40	VD
**3Y-TZP-2-wt% SnO**_**2**_	0,32	980±40	GBD
**PMM8-3Y-TZP**	0,32	804±40	GBD
**PMM8-3Y-TZP-2 wt% SiO**_**2**_	0,52	662±40	VD
**PMM8-3Y-TZP-0,2 wt% SnO**_**2**_	0,51	648±40	VD
**PMM8-3Y-TZP-2 wt% SnO**_**2**_	0,58	560±40	VD

The sintering kinetics was found to be different with each methods of synthesis. The sintering mechanism was observed to change from volume diffusion (VD) to grain boundary diffusion (GBD) in nanopowders 3Y-TZP with the addition of 0.2 and 2 wt% silica obtained by co-precipitation. As for the SnO_2_ additive, the addition of 0.2 wt% was not enough for the sintering mechanism to change, as a higher additive concentration is necessary in this case and, as can be seen from Fig 8, the mechanism changed at the addition of 2 wt% SnO_2_.

**Fig 10:**
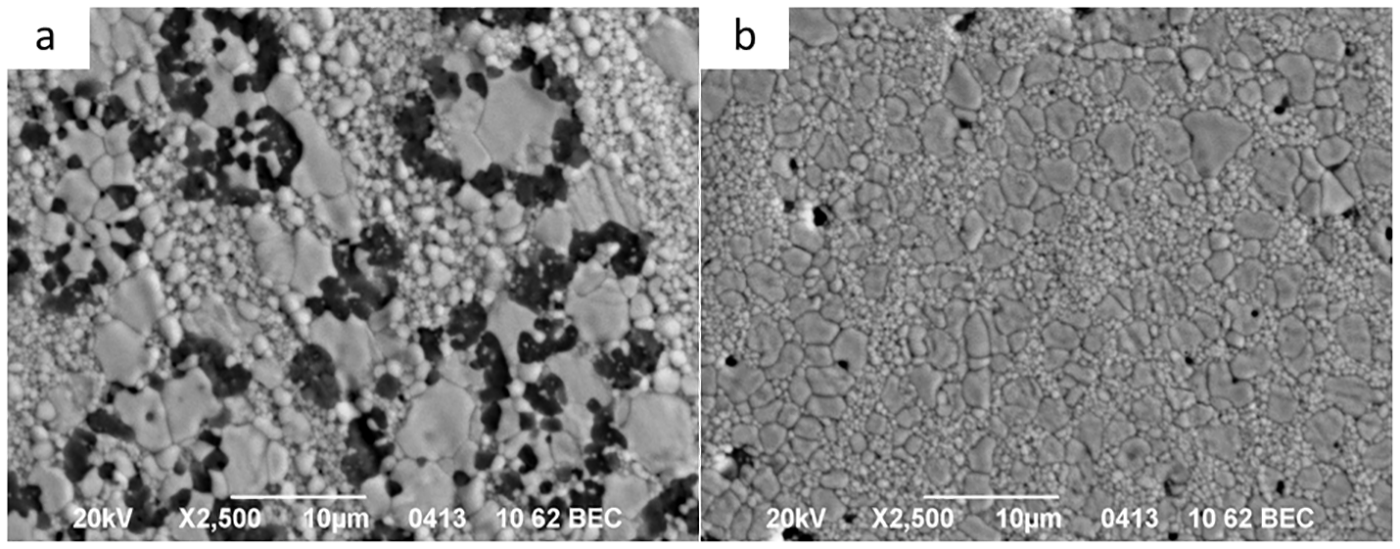
The SEM images of (a) 3Y-TZP-2 wt% SiO_2_ and (b)3Y-TZP-2 wt% SnO_2_ sintered ceramics.

Due to the finding that the silica additive had the most significant influence on the kinetics of the initial sintering stage, the effects of silica additive were considered in detail and are presented in Fig 10.

**Fig 11:**
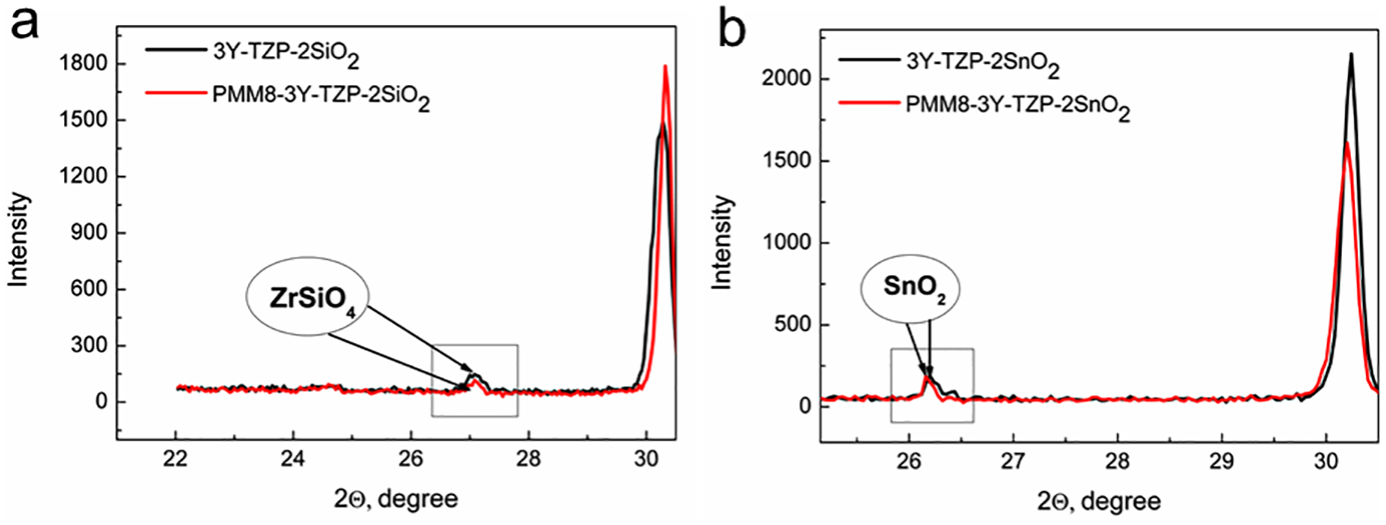
The XRD patterns of (a) PMM8/3Y-TZP-2 wt% SiO_2_ and (b) PMM8/3Y-TZP-2 wt% SnO_2_ sintered ceramics.

Using the co-precipitation method, the change in the diffusion mechanism was caused by solid solution decomposition that occurred with the increasing temperature during sintering and with the beginning of zircon formation. The dopant distribution (Si^4+^) in the initial composition of the solid solution 3Y-TZP-SiO_2_ was changed because this amount (0.2 and 2 wt% silica) was not enough for ZrSiO_4_ formation (the SiO_2_ amount should increase to 30%). The Si^4+^ ions diffused in the zirconia matrix and formed the ZrSiO_4_ grains. The Si^4+^ ions diffusion path was directed to zirconia grain boundaries, and flower like ZrSiO_4_ grains were observed by TEM between the zirconia grains (Fig 10a). The SnO_2_ and SiO_2_ in 3Y-TZP as separated phases were also observed by XRD method (Fig 11).

This fact confirms that the SiO_2_ solution limit in ZrO_2_ lattice is at a level near 0.5 wt%. This is the reason for the predominance of GBD at the initial sintering stage in co-precipitated nanopowders. The sintering mechanism change is accompanied by an increase in activation energy (Table 2).

**Fig 12:**
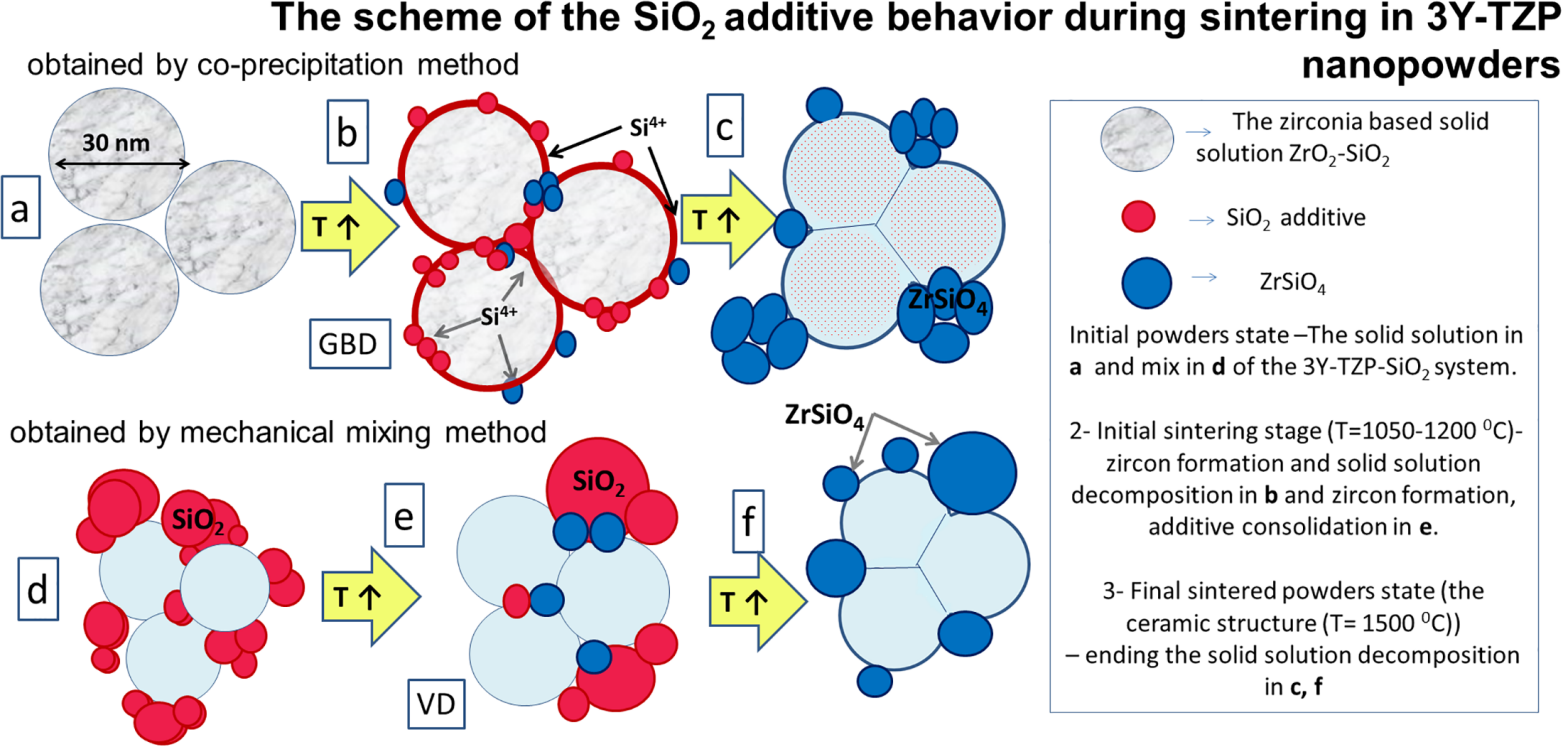
The scheme of the silica additive influence on the sintering process of 3Y-TZP nanopowder.

**Fig 13:**
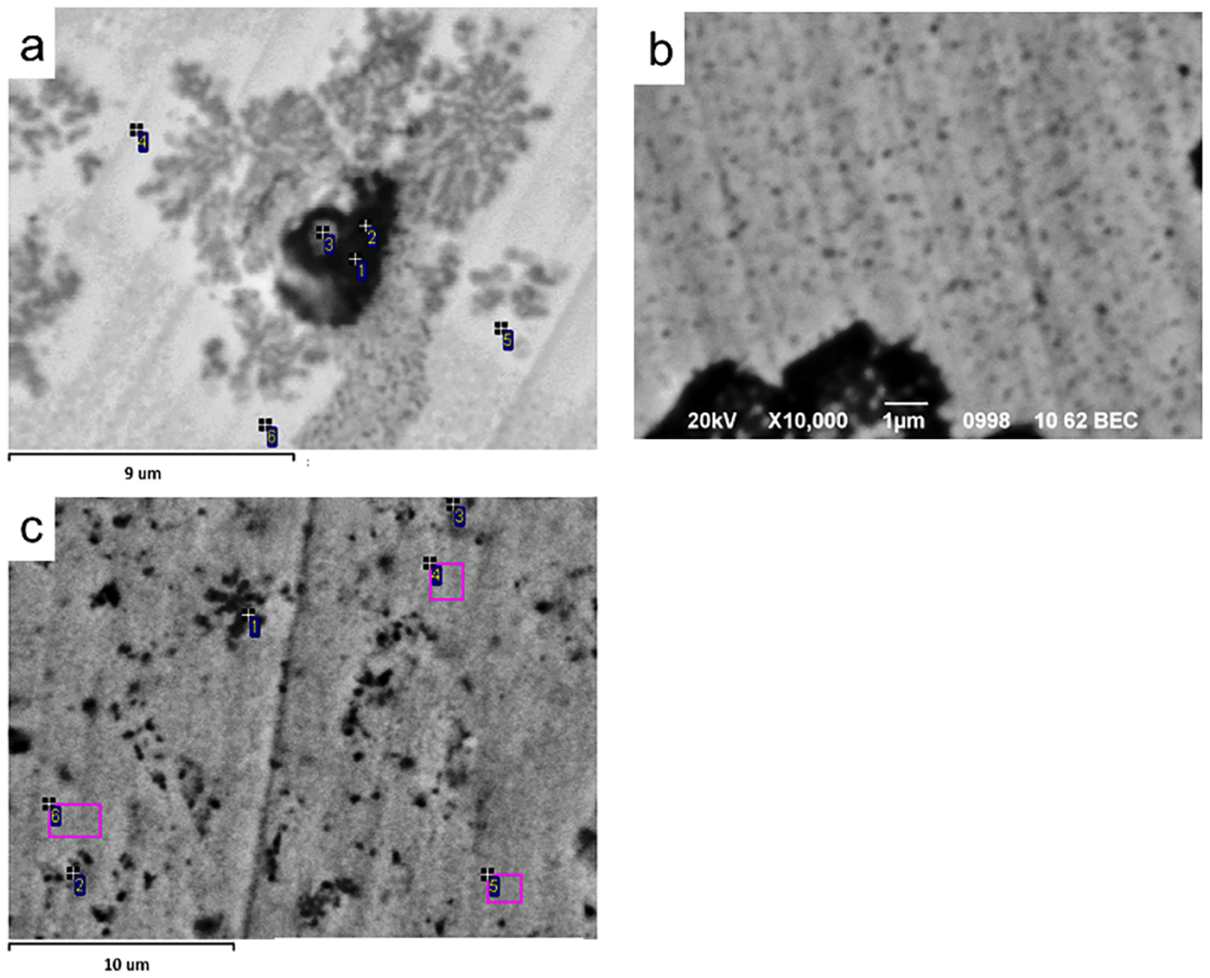
The SEM images of sintered samples (a, b) 3Y-TZP-2 wt% SiO_2_ and (c) PMM8-3Y-TZP-2 wt% SiO_2_.

In the second case, (Fig 12) the behaviour of the silica during sintering obtained by the mixing method is shown. As was found earlier, the solid solution was not created and only zircon formation on zirconia grain boundaries was observed without diffusion of Si^4+^ ions from zirconia grains to the boundaries (Fig 12e). This was confirmed by SEM investigations (Fig 13c). In this case, the Si^4+^ in ZrO_2_ grains was detected at less than a 0.2 wt% level (in recalculation to SiO_2_).

It should be noted that the remaining Si^4+^ in ZrO_2_ grains accounts for 0.3–0.6 wt% (in recalculation to SiO_2_). In Fig 13a, the diffusion pass of Si^4+^ ions can be seen in the white zone (with low Si^4+^ content) in the back-scattering electron image. The black zone on this image represents ZrSiO_4_ grains, the grey zone represents ZrO_2_ grains and white grains are zirconia grains with low Si^4+^ content (Fig 13b).

**Fig 14:**
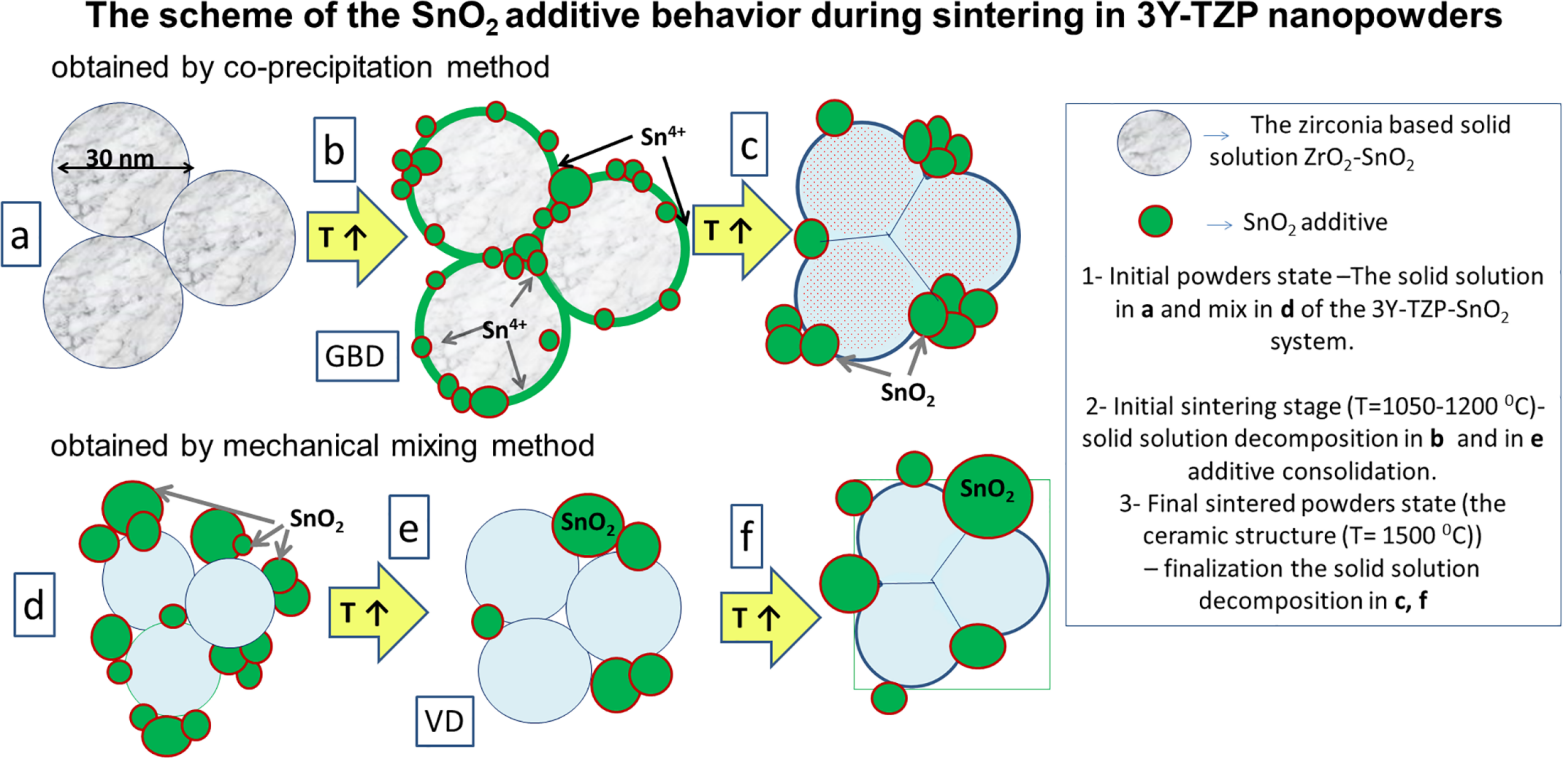
The scheme of the tin oxide additive influence on the sintering process of 3Y-TZP nanopowder.

The scheme of the SnO_2_ additive behaviour is shown in the Fig 14. As can be seen the SnO_2_ in case of using co-precipitation obtaining method create a solid solution with matrix of zirconia. In contrast to the SiO_2_ additive behavior the SnO_2_ did not create any chemical compounds with ZrO_2_ during heating and just leaved the solid solution. In case of addition of SnO_2_ by mixing method the same behavior during sintering process as silica (Fig 11b) was observed (Fig 14e).

The ceramic structures (SEM images) are shown in Fig 13 and confirm the data shown in Fig 12. As can be seen, the silica is distributed in the 3Y-TZP structure in a different way. This is caused by the synthesis method and the zircon formation. In the case of the tin oxide addition, the same additive’ behaviour was observed, but tin does not create compounds with zirconia and just leaves the solid solution composition, distributed at the grain boundaries and at the triple junctions as a separate phase. This behaviour of the tin oxide addition is completely predicted, so in the literature data there is no mention that tin is capable of forming chemical compounds with zirconium.

Based on the results of the data obtained from the X-ray diffraction analysis, TEM, and dilatometry analysis, the change in the sintering mechanism can be better explained by the larger contribution of the SiO_2_ and SnO_2_ diffusion occurring on the boundary of the grain zirconia (that caused GBD mechanism domination in co-precipitated powders) than by the diffusion of zirconium, which would not agree with the results of other authors [12–18, 25]. In the case of nanopowders obtained by the mixing method, the sintering acceleration effect is caused by a combination of two factors—additives and mechanical activation. Additives are consolidated and transfer from the grain boundaries to the locations of triple junctions where they fill the pore space between the zirconium grains, leading to the predominance of the VD mechanism at the initial stage of sintering.

## Conclusion

The comparative analyses of SiO_2_ and SnO_2_ additives and their concentrations as well as various methods of additives addition impact on the kinetics of the initial sintering stage of tetragonal zirconia nanopowders were investigated. The following results were obtained:

1. It was demonstrated that the SiO_2_ and SnO_2_ additives influenced on the nanopowders’ structure in different ways due to the difference in the obtaining methods and additive types. The additive with the most significant influence on the powders’ 3Y-TZP structure was observed in case of silica obtained by co-precipitation.
2. The SiO_2_ and SnO_2_ additives were the reason for the change in the dominant sintering mechanism from VD to GBD in 3Y-TZP nanopowders obtained by the co-precipitation method. This sintering mechanism changing was not detected previously in literature data. It links with formation of supersaturated solid solutions additives (Si^4+^ and Sn^4+^ in zirconia lattice) with ZrO_2_ during nanopowders synthesis and their decomposition during sintering process.
3. It was found the difference between the SiO_2_ and SnO_2_ influence on the diffusion mechanisms at the initial sintering stage of 3Y-TZP. It was determined that VD to GBD mechanism changing was initiated by 0.2 wt% SiO_2_ but for the same sintering mechanism changing in 3Y-TZP was necessary higher amount of SnO2—2 wt%.
4. It was shown that the SiO_2_ and SnO_2_ dopants addition by mechanical mixing have a contrary influence on the sintering mechanisms at the initial stage. In this case the GBD in initial mixed 3Y-TZP nanopowder changed on VD with SiO_2_ and SnO_2_ dopants addition that have a good agreement with literature data.

## Abbreviations

3Y-TZP: 3 mol % yttria-stabilized tetragonal zirconia polycrystal
CRH: constant rate of heating
DIPE: Donetsk Institute for Physic and Engendering
GBD: grain-boundary diffusion
NAS: National Academy of the Science
PMM4 and PMM8: powder mixing and milling during for 4 and 8 hours
S_BET_: specific surface area measured by the Brunauer-Emmett-Teller (BET) method
TEM: Transmission electron microscopy
VD: volume diffusion
XRD: X-ray diffraction
